# Biomechanical assessment of ten fixation techniques after sagittal split ramus osteotomy for significant mandibular advancement

**DOI:** 10.4317/medoral.27625

**Published:** 2025-10-17

**Authors:** Willian Saranholi da Silva, Rogério Leone Buchaim, Constantinos Laskarides, Daniel Oreadi, Archana Viswanath, Beethoven Estevao Costa, Maísa Pereira-Silva, Osvaldo Magro Filho, Paulo Domingos Ribeiro-Junior

**Affiliations:** 1 Department of Oral and Maxillofacial Surgery, Sagrado Coracão University (USC), Bauru, SP, Brazil.; 2 Department of Oral and Maxillofacial Surgery, Faculty of Dentistry of Bauru, University of São Paulo University (FOB-USP), Bauru, São Paulo, Brazil; 3 Department of Oral and Maxillofacial Surgery, Tufts University, Boston, Massachusetts, USA; 4 Department of Oral &amp; Maxillofacial Pathology, Radiology and Medicine, New York University (NYU), New York, New York, USA; 5 Department of Diagnosis and Surgery, School of Dentistry, São Paulo State University (UNESP), Araçatuba, SP, Brazil

## Abstract

**Background:**

This study aimed to biomechanically evaluate ten different fixation methods following SSRO, simulating a 10mm mandibular advancement, with variations in plate position, angulation, and the use of bicortical screws.

**Material and Methods:**

Fifty polyurethane hemimandibles were randomly assigned to ten groups (n=5). SSRO was performed and stabilized using different configurations of 2.0mm plates and screws. Fixation methods included monocortical plates in varying angulations and positions, dual-plate systems, and hybrid techniques incorporating bicortical screws. All specimens underwent three-point compression testing using a universal testing machine. Peak compressive force and displacement were recorded.

**Results:**

The best biomechanical performance was observed in the group using two straight plates placed laterally at a +20° angle (Group 5). Groups employing hybrid or dual-plate systems performed significantly better than those using a single plate. Bicortical screws reduced horizontal displacement but were less effective alone in resisting vertical compressive forces.

**Conclusions:**

Dual-plate fixation with specific angulation improves resistance to compressive forces in large mandibular advancements. The use of a single plate is not recommended unless combined with bicortical screw support.

## Introduction

The sagittal split ramus osteotomy (SSRO) remains one of the most widely used techniques for the correction of dentofacial deformities (DF) (1 , 2). The search for an ideal fixation system following SSRO has been the focus of numerous studies (3 , 4), aiming to achieve stable outcomes while minimizing complications such as occlusal relapse and temporomandibular joint (TMJ) disorders (5 , 6).

The increasing number of patients with severe functional and aesthetic impairments-particularly those with obstructive sleep apnea or craniofacial syndromes-has led to a greater demand for mandibular advancement procedures (7 , 8). In such cases, SSRO is often employed to achieve significant mandibular advancements, improving upper airway patency and facial harmony (9 , 10).

Technological advances have led to the development of more predictable and rigid fixation systems (11 , 4). Long, thick plates and bicortical screws have been shown to enhance segment stabilization and promote early bone healing (12). However, excessive rigidity can sometimes result in adverse effects such as occlusal disharmony, bone compression, failure of osteosynthesis, or undesired torque on the mandibular condyle (6).

Biomechanical and finite element studies have demonstrated the superior stability provided by bicortical screws in SSRO (13 - 16). Nonetheless, their use carries potential risks to adjacent anatomical structures, including dental roots, nerves, and vascular bundles (13). Various plate configurations have also proven effective in promoting stability during mandibular advancement (17 - 20).

A recent study by Klein et al. (17) proposed the use of two straight plates as a reliable option for large advancements. However, biomechanical, photoelastic, and finite element data addressing the performance of osteosynthesis systems under extreme mandibular movements remain scarce.

Segment stability depends on multiple local factors, including the type of fixation used, the quality and quantity of bone, occlusal stability, and the specific position of the fixation device (21 , 6). In clinical scenarios where ideal placement is compromised by anatomical constraints or limited bone availability, alternative plate positioning may be required.

The lack of evidence regarding the biomechanical consequences of using fixation plates in non-ideal positions after large mandibular advancements through SSRO motivated the present study.

## Material and Methods

Biomechanical Testing Setup

A total of 50 polyurethane hemimandibles (Model 1337-3; Sawbones, Vashon, WA, USA), simulating the mechanical properties of human bone with cortical and medullary layers, were used. The samples were randomly assigned to ten groups (n=5), based on previous sample size calculations from similar studies (22 - 24).

A standardized sagittal split ramus osteotomy was performed on each hemimandible using a template-guided approach, following the modified technique proposed by Epker (25). A mandibular advancement of 10mm was achieved in all specimens using custom-made acrylic guides.

Fixation was performed using 2.0mm system titanium plates (1.0mm thickness) and monocortical screws (2.0×5.0mm). In two groups, additional bicortical screws (2.0×13mm) were employed (Neoface System 2.0mm; NeoOrtho, Curitiba, Brazil).

Plates were placed in three spatial orientations relative to the occlusal plane: Parallel (0°), angled negatively (20°), or positively (+20°). The distribution of experimental groups is summarized below:

Group 1: One straight plate (two screws per side), parallel to the occlusal plane.

Group 2: One straight plate, 20° angulation.

Group 3: One straight plate, +20° angulation.

Group 4: Two straight plates, one near the osteotomy and one at the mandibular base, both parallel.

Group 5: Two straight plates at +20° angulation.

Group 6: Two straight plates, both laterally distant from each other.

Group 7: Two plates placed close together at the superior lateral surface.

Group 8: Two plates placed close together at the basal lateral surface.

Group 9: One straight plate + one retromolar bicortical screw.

Group 10: One semi-curved plate with three non-linear screws + one retromolar bicortical screw.

**Figure 1:**
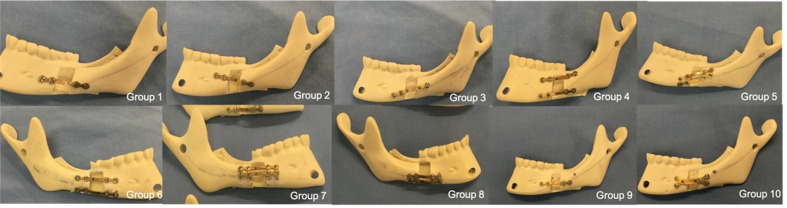
Hemimandibles featuring sagittal osteotomy of the mandibular ramus, with various osteosynthesis methods applied for biomechanical testing.

A positioning template in acrylic resin was used to ensure uniform placement of the fixation devices in all samples. (Figure 1)

1

Biomechanical Test Protocol

**Figure 2:**
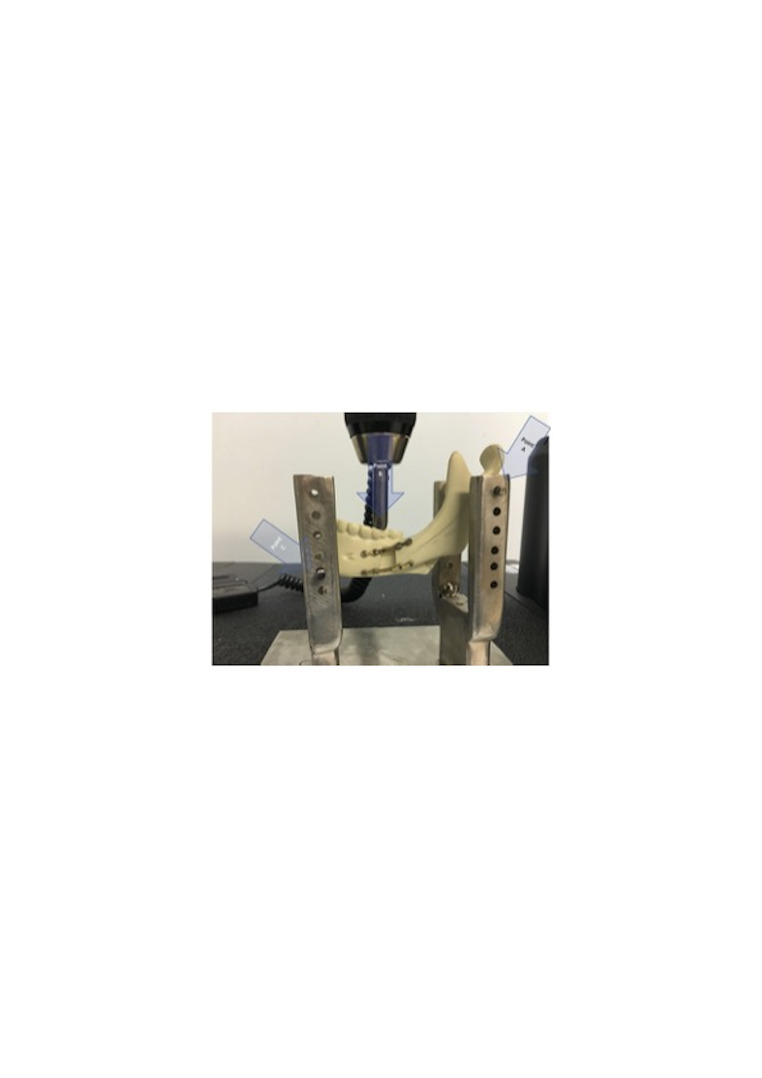
Point A: Represents the resistance provided by the mandibular condyle. Point B: Represents the resistance provided by the food bolus. Point C: Represents the resultant of the masticatory muscle forces.

All tests were performed in randomized order using a universal testing machine (Model 4202; Instron, Norwood, MA, USA), configured for a three-point compression test (22 , 24). The hemimandibles were fixed on a custom metallic support base designed to simulate masticatory force vectors (23). The posterior region (point A) simulated condylar support, while the load cell applied compression to the occlusal surface (point B), with a reaction point at the anterior mandible (point C), mimicking muscle force distribution. (Figure 2)

2

**Figure 3:**
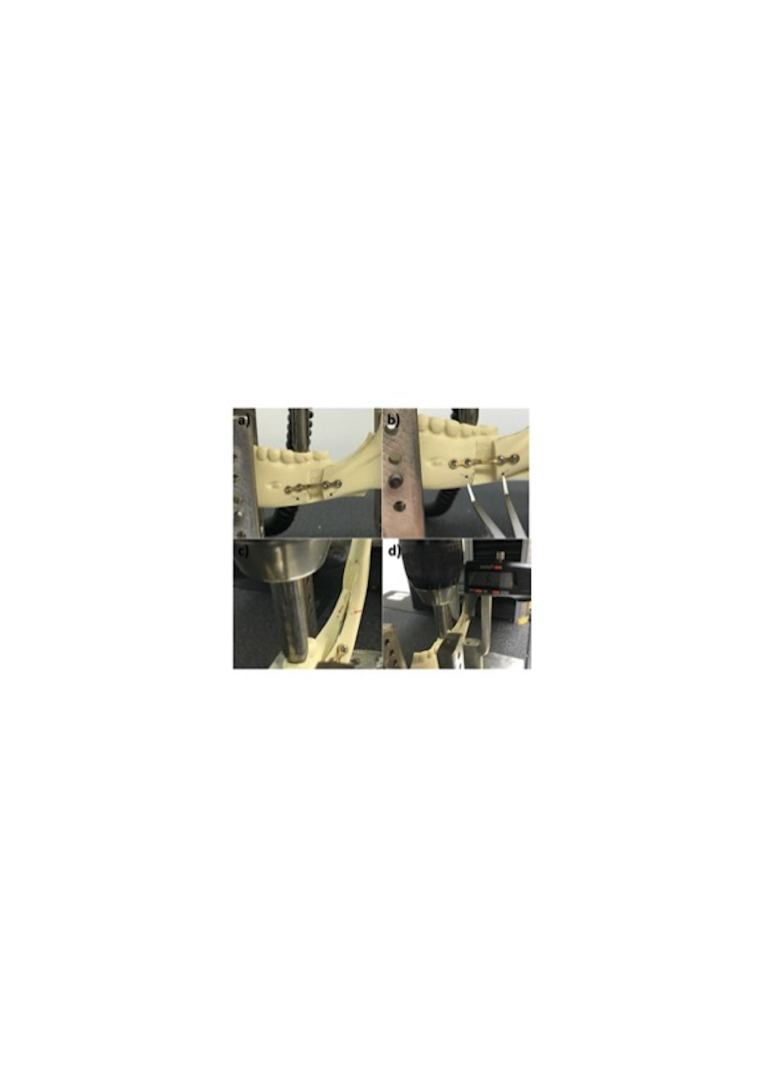
Displacement Measurement Setup: a) Pre-drilled reference cavities on the inferior mandibular border for assessing anteroposterior movement. b) A surgical caliper used to accurately record displacement measurements. c) Pre-drilled reference cavity.

The compression force was applied vertically at a rate of 1mm/min until a 3mm displacement was observed between segments. Displacement was recorded in two regions: (1) The inferior mandibular border for anteroposterior movement and (2) the retromolar area for lateral displacement. These were measured using pre-drilled reference cavities, a surgical caliper, and a digital vernier caliper before and after testing. (Figure 3)

3

The three-dimensional behavior of the osteosynthesis systems was thus evaluated under simulated functional loading conditions.

Statistical Analysis

Data were analyzed using Minitab® 19 (Minitab Inc., State College, PA, USA). Descriptive statistics and exploratory analysis were performed, followed by one-way ANOVA to assess group differences. The assumptions of normality, homoscedasticity, and independence were verified. Tukey's post hoc test was used for multiple comparisons between groups. A significance level of p&lt;0.05 was adopted.

## Results

Biomechanical Analysis

All hemimandibles (HMs) were subjected to linear compression testing, and the peak force values at 3mm displacement were recorded. Preliminary analysis of the assumptions for ANOVA led to the exclusion of one outlier from Group 5, identified as a highly discrepant residual in the graphical residual analysis (Table 1).

Table 1

**Figure 4:**
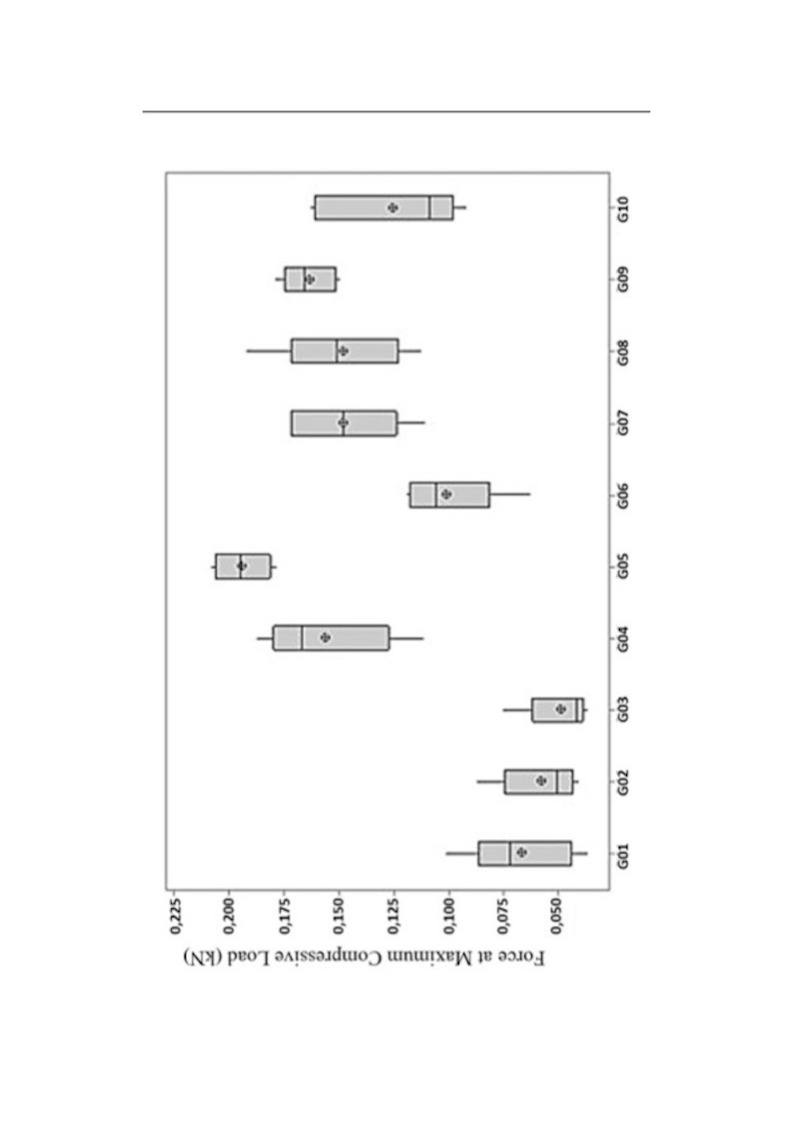
Comparative box plots of peak compressive force (kN) by group.

Descriptive statistics were calculated for each group, including mean, standard deviation, median, minimum and maximum values, and interquartile ranges (Table 2). All variables, except standard deviation, are represented in box plots (Figure 4).

Table 2 4

Groups 5 and 9 demonstrated the highest peak force means and lowest variability, suggesting superior biomechanical stability. Conversely, Groups 1, 2, and 3 displayed the lowest peak force values, indicating reduced mechanical performance under loading. (Figure 4).

A one-way ANOVA was performed to determine whether the type of osteosynthesis significantly influenced the compressive strength. The null hypothesis of equal group means was rejected (F=21.45, p&lt;0.001), confirming a statistically significant effect of osteosynthesis method on load resistance (Table 3). F(9, 39)=21.45, p&lt;0.001.

Table 3

To identify statistically distinct groupings, Tukey's post hoc test was applied, maintaining a familywise confidence level of 95% (26 , 27). Group 5 showed no statistical difference from Groups 4, 7, 8, and 9 but differed significantly from Groups 1-3, 6, and 10 (Table 4).

Table 4

## Discussion

The methods of osteosynthesis used in orthognathic surgery (OS) remain the subject of ongoing debate. In cases involving large dentofacial deformities-particularly those associated with obstructive sleep apnea or syndromes-significant mandibular advancements are often necessary. To achieve predictable and effective outcomes, it is essential to consider both the positioning and type of fixation system used in these procedures.

This preclinical study aimed to evaluate the compressive strength provided by ten different osteosynthesis configurations used in the fixation of sagittal split ramus osteotomies (SSRO) with a standardized 10mm advancement. The influence of plate location, angulation, number, and the addition of bicortical screws were biomechanically assessed.

The decision to test various fixation positions reflects the frequent clinical need to place plates outside of the anatomically ideal zone. Factors such as anatomical asymmetries, bone atrophy, atypical fractures, intraoperative limitations, proximity to the mandibular canal, dental presence, and limited bone thickness often restrict optimal placement. According to the principles of the AO/ASIF, osteosynthesis plates should be placed parallel to the occlusal plane to neutralize compression and tension forces (22). However, whether this applies to large mandibular advancements remains unclear. This study therefore evaluated alternative positions for fixation.

Biomechanical testing showed that Group 5 (two closely spaced plates at +20° to the occlusal plane) yielded the highest mean compressive strength, although it did not differ statistically from Groups 4, 7, 8, and 9. Groups 10, 6, 1, 2, and 3 performed significantly worse than Group 5. Notably, improved performance was observed when plates were placed close together: Group 5 (angled), Group 8 (parallel, at the basal lateral region), and Group 7 (upper region of the SSRO). These configurations yielded outcomes comparable to the classical parallel placement technique tested in Group 4, in line with findings from Klein et al. (23).

All two-plate configurations outperformed single-plate ones in compressive resistance. Despite the relatively poor performance of Group 6, which also employed two plates, its results still exceeded those of single-plate groups. These findings support previous studies that showed greater stability with double-plate systems for both small (24 , 25) and large advancements (23).

The use of bicortical screws in Groups 9 and 10 produced mixed outcomes. While Group 9 (one plate + bicortical screw) outperformed Group 10 (semi-curved plate + bicortical screw), both configurations prevented horizontal displacement of mandibular segments-suggesting enhanced transverse stability. These findings contrast with previous studies involving smaller advancements, where bicortical fixation alone yielded superior resistance (26).

The plate placement in Groups 3 and 5, angled at +20° to the occlusal plane, resembles the Champy technique for mandibular angle fracture fixation. Although SSRO and angle fractures differ biomechanically (27), the similarity is noteworthy. Both preclinical and clinical studies have reported favorable outcomes with the Champy method (26 , 28).

Champy's original concept (7) is widely accepted for angle fracture management and has demonstrated low complication rates in various studies (1 , 11 , 12 , 22). In this study, Group 3 (one plate, Champy angle) showed limited resistance; however, when a second plate was added at the same angle and location (Group 5), results improved substantially-challenging the notion that fixation plates must always remain parallel to the occlusal plane.

While promoting segment stability, osteosynthesis should also reduce postoperative complications. Previous studies associated basal plate placement with increased complication rates (2). However, in this study, Group 8, which featured two basal plates placed close together, performed well biomechanically-suggesting that clinical complications may not arise solely from mechanical instability.

Fixation methods in SSRO are typically classified into three types: Bicortical screws, monocortical miniplates, and hybrid techniques. In vitro studies comparing these methods have yielded heterogeneous results, indicating a need for greater methodological standardization (16). Still, a consensus exists favoring the 3mm displacement threshold as a clinically relevant outcome over peak load testing, since postoperative patients are generally limited to a soft diet and apply lower masticatory forces during early healing (16 , 23).

Consistent with previous findings (23 , 26 , 29 , 30), the weakest configurations in this study involved a single miniplate with four to six monocortical screws (Groups 1-3). In contrast, the hybrid technique used in Group 9 (one plate + bicortical screw) produced the second-best performance, making it a viable option when limited anatomical access precludes the use of two plates.

The present study addresses a gap in the literature identified by Kuik et al. (16), emphasizing the need for in vitro research on large mandibular advancements. All ten fixation configurations in this study were tested under standardized conditions with 10mm advancements-an experimental model rarely addressed in previous studies.

## Conclusions

In conclusion, this biomechanical evaluation of sagittal split ramus osteotomy (SSRO) fixation techniques for large mandibular advancements (10mm) revealed key insights. The optimal outcome was observed in Group 5, which used two straight miniplates at a +20° angle to the occlusal plane with eight monocortical screws, offering the highest resistance to compressive forces.

Single-plate fixation systems performed poorly on their own but showed improved stability when combined with a retromolar bicortical screw in a hybrid technique, as seen in Group 9. Importantly, a single monocortical miniplate is not recommended for large advancements due to inadequate stability. These findings highlight the importance of plate positioning, angulation, and hybrid configurations in enhancing SSRO stability during extensive mandibular movements.

## Figures and Tables

**Table 1:** Table Experimental peak compressive force (kN) for each hemimandible group. (Table inserted with raw values and outlier indicated).

Group	Force atm Maximum Compressive Load (kN)				
	M1	M2	M3	M4	M5
1	0,101	0,072	0,037	0,052	0,072
2	0,087	0,046	0,041	0,051	0,062
3	0,037	0,041	0,042	0,075	0,049
4	0,143	0,173	0,167	0,187	0,112
5	0,189	0,179	0,201	*	0,208
6	0,119	0,1	0,106	0,117	0,063
7	0,137	0,172	0,148	0,172	0,111
8	0,151	0,113	0,152	0,192	0,133
9	0,166	0,171	0,153	0,15	0,179
10	0,092	0,109	0,104	0,159	0,163

1

**Table 2:** Table Descriptive statistics for peak compressive force (kN) across groups. (Insert with mean, SD, Q1, median, Q3, min/max per group).

Group	Count	Mean	SD	Minimum	Q1	Median	Q3	Maximum Value
1	5	0,067	0,024	0,037	0,044	0,072	0,086	0,101
2	5	0,057	0,018	0,041	0,043	0,051	0,074	0,087
3	5	0,049	0,015	0,037	0,039	0,042	0,062	0,075
4	5	0,156	0,029	0,112	0,128	0,167	0,18	0,187
5	4	0,194	0,013	0,179	0,181	0,195	0,206	0,208
6	5	0,101	0,023	0,063	0,082	0,106	0,118	0,119
7	5	0,148	0,026	0,111	0,124	0,148	0,172	0,172
8	5	0,148	0,029	0,113	0,123	0,151	0,172	0,192
9	5	0,164	0,012	0,15	0,151	0,166	0,175	0,179
10	5	0,125	0,033	0,092	0,098	0,109	0,161	0,163

Q1: Median. Q3: Minimum and maximum Force at Maximum Compressive Load (kN) for each of the treatments. Q1: First quartile. Q3: Third quartile.

**Table 3:** Table ANOVA results for peak compressive force (kN).

Source	GL	SQ (Aj.)	QM (Aj.)	F Value	P-Value
Group	9	0,1072	0,0119	21,45	0,000§
Error	39	0,0217	0,0006		
Total	48	0,1289			

GL, degrees of freedom. SQ, sum of squares. QM, mean square. F value, ANOVA statistic. Note: §Significant difference when p<0.05. Confidence level: 95%.

**Table 4:** Table Tukey multiple comparisons of group means (α=0.05).

Group	N	Mean	SD			Grouping		
5	4	0,194	0,013	A				
9	5	0,164	0,012	A	B			
4	5	0,156	0,03	A	B			
8	5	0,148	0,029	A	B	C		
7	5	0,148	0,026	A	B	C		
10	5	0,125	0,033		B	C		
6	5	0,101	0,023			C	D	
1	5	0,067	0,024				D	E
2	5	0,057	0,018				D	E
3	5	0,049	0,015					E

Means that do not share a letter are statistically different (p<0.05).

## Data Availability

The datasets used and/or analyzed during the current study are available from the corresponding author.
